# Peer and familial influences on the association between behavioral inhibition and trajectories of social anxiety symptoms across adolescence

**DOI:** 10.1111/jora.70194

**Published:** 2026-05-15

**Authors:** Madison Politte‐Corn, Sarah Myruski, Kristin A. Buss

**Affiliations:** ^1^ Department of Psychology The Pennsylvania State University University Park Pennsylvania USA; ^2^ Department of Human Development and Family Studies The Pennsylvania State University University Park Pennsylvania USA

**Keywords:** adolescents, behavioral inhibition, family, friends, peers, social anxiety

## Abstract

Behavioral inhibition (BI) is one of the most reliable predictors of social anxiety, and both peers and family members play a key role in shaping this association. In a sample of adolescents enriched for elevated BI and social anxiety symptoms, we examined (1) the moderating role of familial and close peer relationship qualities on associations between BI, concurrent social anxiety symptoms, and social anxiety symptom trajectories and (2) indirect effects of adolescent BI on social anxiety through close peer and familial relationship qualities. Furthermore, we examined sex differences in these pathways. When adolescents were 12–14 years old (*N* = 150; 60% female at birth; 80% White; 38% annual family income <$70,000), parents reported on BI and youth self‐reported familial and close peer relationship qualities. In addition, adolescents self‐reported social anxiety symptoms annually across 4 years. Multiple‐group growth curve models indicated that, for adolescent girls, perceived support from close peers interacted with BI to predict both concurrent social anxiety and social anxiety symptom trajectories. For adolescent boys, perceived familial negative interactions interacted with BI to predict social anxiety symptom trajectories. Finally, for adolescent girls, BI indirectly predicted social anxiety through low perceived support from family members, but not through peer relationship qualities. Results provide support for social risk and protective factors that shape anxiety trajectories across this vulnerable developmental period for BI youth.

## INTRODUCTION

Behavioral inhibition (BI) is an early‐emerging, relatively stable temperamental profile characterized by heightened fear and avoidance of novelty (García‐Coll et al., 1984). Critically, BI is one of the most reliable predictors of internalizing symptoms, with meta‐analytic data indicating that over 40% of behaviorally inhibited children develop social anxiety disorder (SAD) as adolescents (Clauss & Blackford, 2012) and more experience subclinical levels and symptoms related to social anxiety. Yet, a large proportion of BI children do not develop clinically significant social anxiety symptoms, raising questions about other factors that may shape risk or resilience for SAD. Importantly, prospective‐longitudinal studies suggest that the onset of SAD often occurs between ages 10 and 17 (Magee et al., 1996; Wittchen & Fehm, 2001), a developmental period characterized by social re‐orientation from parents to peers as the prominent social context (Larson & Richards, 1991). As such, both familial and peer relationships in adolescence likely play a role in shaping developmental trajectories from temperamental risk to SAD.

### The association between BI and social anxiety

The term “behavioral inhibition” was first coined by Cynthia García‐Coll and Jerome Kagan to describe a heightened vigilance and lack of approach to novelty in toddlers (García‐Coll et al., 1984). In subsequent work, Kagan et al. (1999) demonstrated that approximately 46% of infants who exhibited heightened fearful behavior in response to novelty showed anxious symptomatology at age 7. Schwartz et al. (1999) showed that BI in toddlerhood is uniquely associated with social anxiety symptoms relative to other forms of internalizing in adolescence, and this specificity to SAD risk was supported by later work (Bourdon et al., 2019; Hirshfeld‐Becker et al., 2007).

Subsequent work in other samples has substantiated the robust predictions from early BI to later SAD or broader impairments in social functioning (e.g., Biederman et al., 2001; Chronis‐Tuscano et al., 2009; Hirshfeld‐Becker et al., 2007). For instance, BI in toddlerhood was positively related to social reticence across ages 2–5 (Degnan et al., 2014) and predicted a high, stable trajectory of socially anxious behavior during a speech task across ages 5 to 13 (Poole et al., 2022). Furthermore, using the same sample, Chronis‐Toscano et al. demonstrated that children who were classified as behaviorally inhibited at age four were 3.79 times more likely to meet criteria for a diagnosis of SAD in adolescence (Chronis‐Tuscano et al., 2009), and this association has been replicated in other studies (Biederman et al., 2001; Hirshfeld‐Becker et al., 2007). The magnitude of the association as well as shared phenotypic measurement of BI and social anxiety symptoms has raised questions about whether BI is simply a prodromal form of social anxiety (Pérez‐Edgar & Guyer, 2014). However, many studies suggest that BI and social anxiety are separate phenotypes with unique biobehavioral profiles (Klein & Mumper, 2018; Politte‐Corn et al., 2025), and social anxiety disorder uniquely includes functional impairment as a necessary component for diagnosis.

### Socio‐contextual risk and protective factors

#### Parents and family

Given heterogeneity in the prediction from BI to social anxiety, researchers have long underscored the important role of the environment in shaping this association (Fox et al., 2022; Kagan & Snidman, 1999). Specifically, caregiving behaviors are considered one of the main moderating factors that either exacerbate or attenuate the development of anxiety in behaviorally inhibited children (see Degnan et al., 2010 and Ryan & Ollendick, 2018 for reviews). For instance, one recent study found that inhibited toddlers who experienced low dismissive and high supportive parenting showed decreasing social anxiety symptoms across ages 9–15 (Lorenzo et al., 2022). At the same time, numerous studies suggest that over‐engaging in traditionally “supportive” parenting practices can maintain or exacerbate social anxiety symptoms for youth at temperamental risk (Kiel et al., 2021; Kiel & Buss, 2014; Rubin et al., 2002; Suarez et al., 2021). For example, inhibited toddlers who received more affectionate/oversolicitous behavior from their mother demonstrated poorer emotion regulation, which in turn predicted more socially anxious behavior in early adolescence (Suarez et al., 2021). Interestingly, oversolicitous parenting may be an important mechanism for boys higher in BI, given prior work showing that parents respond to boys' fearful behaviors with greater concern, intrusiveness, and protective parenting compared with girls (Kiel et al., 2016; Park et al., 1997).

Consistent with the detrimental impact of oversolicitous parenting for inhibited children, numerous studies suggest that familial accommodation of anxiety during adolescence shapes the association between anxiety severity and social impairment, both as a mediator (de Barros et al., 2020; Kagan et al., 2016) and moderator (Etkin et al., 2022) of this effect. Familial accommodation refers to changes in behavior or routine in response to children's anxiety that are intended to prevent or alleviate the child's distress (Etkin et al., 2022; Lebowitz et al., 2012). These behaviors, while intended to be supportive, instead maintain or exacerbate youth anxiety symptoms and associated impairment. Alternatively, other forms of familial support during adolescence appear to be protective against social anxiety (Boulton & Macaulay, 2022; Nelemans et al., 2020). For instance, one study demonstrated that adolescent social anxiety symptoms were inversely associated with mother‐reported autonomy support and positively associated with adolescent‐ and mother‐reported psychological control (Nelemans et al., 2020). Furthermore, parental discussions of challenges with their adolescents indirectly predicted lower social anxiety by increasing adolescents' self‐esteem (Boulton & Macaulay, 2022). These varying impacts of familial support may be explained in part by a recent model of emotion socialization in adolescence, which situates parenting practices along the dimensions of emotional engagement and emotional guidance (Miller‐Slough & Dunsmore, 2016); while both may be perceived as supportive, high emotional engagement in the absence of emotional guidance (e.g., accommodation or co‐rumination) may actually be maladaptive for anxious youth.

Taken together, the extant literature suggests that oversolicitousness and accommodation from caregivers may exacerbate risk for social anxiety (e.g., Lewis‐Morrarty et al., 2012; Rubin et al., 2002; Suarez et al., 2021), while autonomy support and high emotional guidance may be protective against social anxiety (Degnan et al., 2010; Hane et al., 2008; Wood et al., 2003). Familial support may also act as a mediator of the association between BI and social anxiety, given bidirectional associations between oversolicitous/accommodating parenting and youth anxiety symptoms (Buss et al., 2021; Kiel et al., 2021). However, most of this work has been conducted in early childhood, data and less is known about how the familial environment during adolescence may facilitate or protect against the emergence of social anxiety during this high‐risk developmental period, particularly for BI adolescents.

Another dimension of the familial environment that has been largely unexamined in the social anxiety literature is familial negative interactions (e.g., conflict and antagonism). Recent work has demonstrated that exposure to interparental conflict predicted increased risk for social anxiety symptoms in adolescents, and this was mediated by increased loneliness and decreased perceptions of friendship support for both girls and boys (Weymouth et al., 2019). Interestingly, prior work suggests that adolescent girls and boys formulate unique appraisals of interparental conflict, such that girls are less prone to negatively appraise interparental conflict when other familial protective factors (e.g., cohesion) are present, but boys negatively appraise interparental conflict regardless of other protective or vulnerability factors in the family (Fosco et al., 2021). Regarding adolescents' relationship with their parents, past work has shown that adolescent–parent hostility predicted higher adolescent social anxiety through increased compliance with peers, characterized by a tendency to change one's thoughts and behaviors to align with peers and a heightened desire for peer approval (Weymouth & Buehler, 2018). Furthermore, in a sample of early adolescent girls oversampled for fearful/shy temperament, maternal expressions of negative facial affect during a conflict predicted higher adolescent social anxiety symptoms 2 years later (Woody et al., 2022). Taken together, these studies provide preliminary evidence that familial conflict may exacerbate risk for social anxiety in adolescents, but more work is needed to elucidate this potential developmental pathway. Furthermore, examining both familial and peer relationships is paramount to highlighting gaps in current etiological models of social anxiety risk.

#### Peers

Peer relationships become particularly salient during adolescence (Larson & Richards, 1991; Nelson et al., 2016), and social anxiety has been consistently linked with low levels of peer acceptance and/or high levels of peer rejection (e.g., Greco & Morris, 2005; La Greca & Harrison, 2005; La Greca & Lopez, 1998; Tillfors et al., 2012). However, less is known about how adolescents' relationships with their close peers (i.e., friends and romantic partners) may facilitate or prevent the development of social anxiety, particularly for youth at high temperamental risk. Extant work suggests that negative interactions with close peers increase risk for social anxiety, with some studies finding that girls are particularly vulnerable (Pickering et al., 2020) and others finding no gender differences in this association (Flanagan et al., 2008; La Greca & Harrison, 2005). For instance, one study demonstrated that friendship quality, but not quantity of friends, moderated the association between peer acceptance and social anxiety for girls but not boys, such that high negativity within girls' close friendships exacerbated risk for social anxiety (Greco & Morris, 2005). Regarding supportive interactions with close peers, some work suggests that positive qualities in best friendships and the presence of a romantic relationship protect against adolescent social anxiety (La Greca & Harrison, 2005; La Greca & Lopez, 1998). Some work, however, has not found a protective role of positive friendship qualities (Greco & Morris, 2005), and some forms of support from close peers may exacerbate risk for internalizing symptoms for some youth (e.g., Barstead et al., 2018; Rubin et al., 2006; Weymouth & Buehler, 2018). For instance, greater compliance with close peers, characterized by a tendency to change one's thoughts and behaviors to align with peers and a heightened desire for peer approval, was associated with increases in social anxiety symptoms for both girls and boys (Weymouth & Buehler, 2018). Furthermore, similar to potentially detrimental effects of familial support on anxiety risk, perceived support from peers may exacerbate risk when characterized by emotional engagement in the absence of guidance (e.g., in the case of co‐rumination or accommodation; Miller‐Slough & Dunsmore, 2016). It is also important to note bidirectional effects between BI, social anxiety, and close friendship qualities (La Greca & Lopez, 1998; Rubin et al., 2018; Tillfors et al., 2012; Vernberg et al., 1992). Specifically, social anxiety and BI predict lower friendship support (La Greca & Lopez, 1998; Tillfors et al., 2012; Vernberg et al., 1992), suggesting a potential mechanism through which at‐risk youth develop more severe social anxiety symptomatology. As such, one possible pathway from BI to social anxiety is through the indirect effects of peer support and/or perceived negative interactions with close peers.

Taken together, the existing literature suggests that negative interactions with close peers increase risk for social anxiety, while close peer support may increase or decrease risk. Furthermore, these close peer relationship qualities may serve as indirect effects in the association between temperamental risk and social anxiety symptoms. However, this body of literature is limited, and more research is needed to elucidate potential developmental pathways from BI to social anxiety.

### The present study

Supported by the extant literature and gaps in our knowledge of these processes, we propose a conceptual model by which relationship qualities in both the familial and peer contexts may shape the association between behaviorally inhibited temperament and social anxiety symptomatology in adolescence. Specifically, the aims of the present study were to examine (1) the moderating role of familial and close peer relationship qualities on associations between BI, concurrent social anxiety symptoms, and social anxiety symptom trajectories and (2) indirect effects of BI on social anxiety through perceived peer and familial relationship qualities. We hypothesized that negative interactions across familial and peer contexts would exacerbate risk for social anxiety. Furthermore, we hypothesized that BI would indirectly predict later social anxiety through high perceived negative interactions with peers or family. Given mixed evidence regarding the role of support from close peers and family members in shaping social anxiety development, particularly for inhibited youth, we hypothesized interactive and indirect effects of supportive interactions on the BI‐anxiety link but did not make hypotheses about the direction of these effects. Moreover, because familial support at both low and high levels (i.e., oversolicitous/overprotecting parenting) may confer anxiety risk among BI youth, with previous work specifically documenting this curvilinear relation (Kiel et al., 2016), exploratory analyses tested curvilinear effects of perceived familial support on the BI‐social anxiety link. Finally, given evidence of sex differences in the interplay between BI, social context and social anxiety (e.g., Fosco et al., 2021; Kiel et al., 2016; Rose & Rudolph, 2006), we also explored sex differences in each of these pathways.

## MATERIALS AND METHODS

### Participants

Parents of adolescents between the ages of 12–14 were recruited in two regions in a mid‐Atlantic state. Families were recruited from a prior study of temperamentally fearful children and using mailing lists, a local birth record and volunteer database, Facebook advertising targeting parents of teenagers in both geographical regions, and through community partnerships (e.g., Boys and Girls Club) and community outreach events through the study's affiliation with Parents and Children Together community‐university research initiative. Inclusion criteria were ability to read and speak English and no known neurological (e.g., epilepsy) or developmental/genetic (e.g., autism) disorders. Recruitment targeted adolescents across a full range of social anxiety risk, such that enrollment continued until at least 40 adolescents with clinically meaningful social anxiety symptoms (SCARED social anxiety score ≥ 8; Birmaher et al., 1997) were recruited. The final sample was enriched for high levels of BI and anxiety, with 98 (50.3%) adolescents who screened eligible for the study meeting the previously established threshold for elevated BI (total BIQ score ≥ 119; Morales et al., 2017; Suarez et al., 2021) and 82 (41.9%) reporting clinically significant social anxiety symptoms.

Adolescents self‐identified as 80% White, 2% Black, 16% more than one race, and 2% Latinx on a questionnaire. Of the 120 families that elected to report their total annual family income, seven (5.8%) earned $30,000 or less, 39 (32.5%) earned $31,000–$70,000, 20 (16.7%) earned $71,000–$100,000, 34 (28.3%) earned $101,000–$150,000, and 20 (16.7%) earned more than $150,000. Of the 154 families that elected to report parental education levels, 12 (7.8%) reported that both parents had less than a 4‐year degree, 53 (34.4%) reported that one parent had less than a 4‐year degree, and 89 (57.8%) reported that both parents had a 4‐year degree or higher.

### Attrition and power analyses

In total, 195 were screened, 150 provided data at T1, and 96 provided data at at least one follow‐up assessment. Most data loss was due to the COVID‐19 pandemic. The analyzed sample consisted of participants who completed the T1 assessment (60% female at birth; *M*_age_ at T1 = 14.12). A sensitivity power analysis conducted via Gpower 3.1.9.2 indicated that a sample size of 150 was sufficient to detect effect sizes at and above *f* ^2^ = 0.053 (Δ*R* ^2^ = .05) at 80% power and alpha level of .05 in a multiple regression model with nine predictors (consistent with our moderation models). For our mediation models, prior simulation studies have demonstrated that a sample size of 150 is sufficient to detect *a* and *b* paths with small to medium effect sizes (Fritz & MacKinnon, 2007). Finally, although our follow‐up sample was smaller than planned due to the COVID‐19 pandemic, over 50% of participants provided longitudinal data, meeting previously established thresholds for reliably using full‐information maximum likelihood (FIML) to account for missing data (Enders & Bandalos, 2001; Graham, 2009).

There were no differences in sex distribution [*χ* ^2^(1) = .57, *p* = .90], age at screening [*t*(190) = −.15, *p* = .88], race/ethnicity [*χ* ^2^(2) = 3.83, *p* = .15], or family income [*χ* ^2^(15) = 7.69, *p* = .94] between participants who completed T1 measures and those who completed the screener only. Furthermore, there were no differences in sex distribution [*χ* ^2^(1) = 1.02, *p* = .31], race/ethnicity [*χ* ^2^(2) = 3.20, *p* = .20], or family income [*χ* ^2^(15) = 13.14, *p* = .59] between participants who did and did not complete the follow‐up measures. There was a significant age difference between participants who did and did not complete the follow‐up questionnaires, such that participants with missing longitudinal data were slightly older at screening [mean age difference = 3.21 months; *t*(190) = 2.23, *p* = .03, *d* = .31]. Among variables analyzed in the current study, 8.0% of values were missing in cross‐sectional models and 13.63% of values were missing in longitudinal models. Little's MCAR test, which included all study variables and demographic variables, was nonsignificant [*χ* ^2^ (32) = 34.26, *p* = .36], indicating that data did not significantly differ from a missing completely at random pattern and supporting our use of FIML to account for missing data.

### Procedure

The full study protocol is described in detail elsewhere (Buss et al., 2025). Briefly, adolescents and their parents were invited to participate in a longitudinal study consisting of interviews, questionnaires, remote tasks, and three laboratory sessions, all of which were completed annually for four consecutive years (T1‐T4), with the exception of the BIQ which was completed by parents only at screening. Data collection for the variables included in the present study occurred between 2018 and 2025. The current analyses focus on the BIQ, T1 questionnaires related to familial and peer relationships, and T1‐T4 social anxiety symptom scores. We limited our analyses to T1 measures of peer and familial relationship qualities because we were interested in how BI interacts with these socio‐contextual factors to influence social anxiety development during adolescence. As such, for this study, BI and the peer/familial relationship variables were best measured concurrently. The Institutional Review Board at the Pennsylvania State University approved this study.

### Measures

#### Behavioral inhibition

*Behaviorally Inhibited Temperament* was measured at screening using parent report from the *Behavioral Inhibition Questionnaire (BIQ)*. The BIQ (Bishop et al., 2003; see Table 1 for descriptive statistics) is a 30‐item questionnaire to assess child temperament characteristics specifically regarding shyness, fearfulness, and withdrawal. Parents use a 7‐point scale (1 = *Hardly ever*, 7 = *Almost always*) to indicate frequencies of observed behaviors. Parents were instructed to respond “based on how you think your child currently compares with other children around the same age,” and items were adapted to refer to adolescents and developmentally appropriate scenarios. We asked parents to report on their adolescent's current behavioral inhibition under the assumptions that BI is relatively stable across development (Chronis‐Tuscano et al., 2009; Gest, 1997; Rubin et al., 2002) and that contemporaneous reports are more reliable than reporting on temperament traits retrospectively. The BIQ yields three higher‐order scales: Social Novelty (14 items; e.g., *Will happily approach a group of unfamiliar adolescents to join their group*), Situational Novelty (12 items; e.g., *Approaches new situations or activities very hesitantly*), and Physical Challenges (4 items; e.g., *Happily explores new forms of entertainment* [e.g., *video games and board games*]). The BIQ has previously been reliably used with adolescents (Broeren & Muris, 2010). All items on the questionnaire are summed to create a total BI score, with higher scores indicating greater BI (*α* = .96). We focus the current analyses on the total BI score. Internal consistency was confirmed to be excellent in the current sample, as indicated above.

**TABLE 1:** Means, standard deviations, and correlations with confidence intervals.

Variable	*M*	*SD*	Min	Max	1	2	3	4	5	6	7
1. Age (years)	14.12	0.81	12.58	15.92							
2. BI	116.58	33.85	33.00	179.00	.02						
					[−.14, .18]						
3. Familial support	3.45	0.60	1.79	4.62	.00	−.27**					
					[−.17, .17]	[−.42, −.10]					
4. Familial negative interactions	2.05	0.58	1.00	3.92	−.01	.13	−.40**				
					[−.19, .16]	[−.04, .29]	[−.53, −.24]				
5. Peer support	2.92	0.75	1.37	4.65	.06	−.07	.18*	.14			
					[−.13, .24]	[−.25, .12]	[−.01, .35]	[−.05, .32]			
6. Peer negative interactions	1.56	0.58	1.00	3.38	.19*	.16*	−.25**	.39**	−.13		
					[−.00, .37]	[−.03, .34]	[−.42, −.06]	[.21, .54]	[−.32, .06]		
7. T1 Social anxiety	6.87	4.37	0.00	14.00	.20**	.57**	−.30**	.13	−.15	.14	
					[.03, .35]	[.44, .67]	[−.45, −.14]	[−.04, .30]	[−.32, .04]	[−.06, .32]	
8. Social anxiety slope	0.38	1.38	−3.74	5.44	.14	.34**	−.08	.11	−.05	.22**	.55**
					[−.02, .30]	[.20, .48]	[−.25, .09]	[−.06, .28]	[−.23, .14]	[.03, .40]	[.43, .65]

*Note*: Values in square brackets indicate the 95% confidence interval for each correlation. Age reflects participants' age at T1. All peer and familial variables were measured at T1.

* *p* < .10.

** *p* < .05.

#### Social anxiety symptoms

*Social anxiety* symptoms were measured at each timepoint using the *Screen for Child Anxiety‐Related Emotional Disorders‐Child Version (SCARED‐C)*. The SCARED‐C (Birmaher et al., 1997; see Table 1 for descriptive statistics) comprises 41 items assessing self‐reported child anxiety symptoms. Similar to the parent version (SCARED‐P), the SCARED‐C is considered the “gold‐standard measure” for comprehensive assessment of child anxiety symptomatology and consistently indicates valid, reliable, and sensitive findings (Behrens et al., 2019). We selected youth‐reported social anxiety rather than parent‐reported to reduce shared‐informant variance (given that BI was parent‐reported) and to more fully capture adolescents' social anxiety symptoms across contexts. Adolescents rate anxiety‐related cognitions or behaviors (e.g., “*I feel nervous with people I don't know well*”) on a 3‐point Likert Scale ([0] Not True or hardly ever true, [1] Somewhat true or sometimes true, and [2] Very true or often true). All items are summed such that higher scores indicate more anxiety symptoms. Each item loads onto one of five subscales: Panic Disorder or Significant Somatic Symptoms (13 items), Generalized Anxiety Disorder (9 items), Separation Anxiety (8 items), Social Anxiety (7 items), and Significant School Avoidance (4 items). We focus the current analyses on the Social Anxiety Disorder scale (*α* > .91 across timepoints). Cronbach's alpha indicated excellent internal consistency in the current sample, as indicated above.

#### Parent–adolescent relationship qualities

*Parent–Adolescent Relationship Qualities* were measured at the first timepoint using the *Network of Relationships Inventory‐Social Provisions Version (NRI‐SPV)*. The NRI‐SPV (Furman & Buhrmester, 1985; see Table 1 for descriptive statistics) measures adolescent‐reported relationship qualities based on social needs and relational characteristics across various types of personal relationships. This 30‐item questionnaire identifies how relationships bring an adolescent reassurance of worth and companionship, and how they view parents as a secure base. Evaluated relationships include a mother figure, father figure, sibling, and relative. For the current study, we created familial composite measures by averaging scores across these four relationship figures, although the results did not substantively change when examining only maternal or nuclear family relationships. Adolescents use a 5‐point Likert Scale ([1] Little or none, [2] Somewhat, [3] Very much, [4] Extremely much, [5] The most) to rate 30 items assessing supportive or negative interactions. The Supportive Interactions second‐order scale is calculated by averaging items across six subscales assessing Companionship, Instrumental Aid, Intimate Disclosure, Nurturance, Affection, and Reliable Alliance (*α* = .93 across all items and relationships). The Negative Interactions scale is computed by averaging items across two subscales assessing Conflict and Antagonism (*α* = .92 across all items and relationships). We focus the current analyses on the Support and Negative Interactions second‐order scales collected at the first timepoint.

#### Close peer relationship qualities

We additionally used the NRI‐SPV described above to assess supportive and negative interactions between adolescents and their close peers. Adolescents identify one same‐sex friend, other‐sex friend, and romantic partner to report on using the same scales described above. Participants were included in analyses as long as they generated a name for at least one of these relationships. Consistent with our approach to operationalizing parent–adolescent relationship qualities, we focus the current analyses on the Support (*α* = .93 across all items and relationships) and Negative Interactions (*α* = .93 across all items and relationships) second‐order scales collected at the first timepoint.

### Data analytic plan

In line with our first goal, we tested the moderating role of peer and familial relationship qualities (measured at T1) on the relation between BI, concurrent social anxiety symptoms, and social anxiety symptom trajectories. Specifically, using a structural equation modeling (SEM) framework, we computed a multiple‐group growth curve model testing close peer support, familial support, close peer negative interactions, and familial negative interactions as moderators of the relation between BI and (1) the intercept of social anxiety symptoms (i.e., T1 social anxiety) and (2) the slope of social anxiety symptoms across T1‐T4. We first computed an unconditional growth model with no predictors to examine the average trajectory of social anxiety symptoms across participants. Growth coefficients indicated the change that occurred in 1‐year increments beginning at T1. Multiple‐group models were used because this allowed us to examine sex differences in the interaction effects, as indicated by likelihood ratio tests comparing the free versus constrained parameter estimate for each interaction term. Using a SEM framework also allowed us to directly test whether peer or familial effects within the same model were significantly different from each other (e.g., if the interaction between BI and peer support was a significantly better predictor of social anxiety than the interaction between BI and familial support). Predictors included in interaction terms were mean‐centered both in the interaction product and when used as main effect variables. Significant interactions were probed by examining simple slopes and the Johnson–Neyman region of significance with confidence bands. For our second aim to examine indirect effects of BI on social anxiety through perceived peer and familial relationship qualities, we computed an additional multiple‐group path model with the peer and familial relationship variables (again measured at T1) entered as mediators of the effect of BI on social anxiety. We used bootstrapped standard errors based on 1000 samples. Full‐information maximum likelihood was used to account for missing data in all regression models. All analysis code and research materials are available upon request to the corresponding author, and data are available through NIH‐NDA. Descriptive analyses were conducted using SPSS version 29.0.0.0, and growth models were analyzed using the *lavaan* package in R version 4.2.1. This study's data analysis was not preregistered.

## RESULTS

### Preliminary analyses

Data were checked for outliers using the outlier labeling rule, which uses the interquartile range (IQR) to detect outliers (Hoaglin et al., 1986). Any data points falling below the lower fence (Q1–1.5 × IQR) or above the upper fence (Q3 + 1.5 × IQR) were flagged as outliers. One outlier was identified (peer negative interactions = 4.83) and handled with extreme value replacement (i.e., replaced with the closest observed value not flagged as an outlier). After outlier correction, no study variables were significantly skewed. Means and standard deviations of study variables are presented in Table 1. Across familial relationships, negative interactions were highly correlated (*r*s = .22–.41; *p*s < .01) and supportive interactions were highly correlated (*r*s = .39–.62; *p*s < .001). Across close peer relationships, negative interactions were highly correlated (*r*s = .35–.58; *p*s < .01), and supportive interactions were highly correlated (*r*s = .25–.64; *p*s < .01). The high degree of intercorrelations among the family and peer variables lends justification for the use of composite measures described above.

#### Descriptive statistics

We next examined any associations between our study variables, age, and sex. As seen in Table 1, age was cross‐sectionally associated with social anxiety symptoms (*r* = .20) and perceived negative interactions with peers (*r* = .19; *p*s < .10). Furthermore, there was a significant difference in social anxiety symptoms between female (*M* = 7.69, *SD* = 4.14) and male (*M* = 5.34, *SD* = 4.44) adolescents at T1, *t*(140) = −3.18, *p* < .001. There were no other significant associations between age or sex and the study variables.

#### Associations between BI, peer and familial relationship qualities, and social anxiety

Correlations between the study variables are presented in Table 1. As expected, BI was positively associated with social anxiety symptoms at T1 (*r* = .57, *p* < .001) as well as the slope of social anxiety symptoms across the study period (*r* = .34, *p* < .001). There was also a small, marginally significant positive correlation between BI and perceived negative interactions with peers (*r* = .16, *p* = .095). Higher perceived negative interactions with peers were also significantly associated with increasing social anxiety symptoms across the study period (*r* = .22, *p* = .022). Familial support was negatively correlated with BI (*r* = −.26, *p* = .002), social anxiety symptoms (*r* = −.30, *p* < .001 at T1; *r* = −.40, *p* < .001 at T2), familial negative interactions (*r* = −.40, *p* < .001), and peer negative interactions (*r* = −.25, *p* = .01). Peer negative interactions were positively associated with familial negative interactions (*r* = .39, *p* < .001), and there was a small, marginally significant positive correlation between perceived familial support and perceived peer support (*r* = .18, *p* = .064). No other study variables were significantly correlated.

#### Unconditional growth models

An unconditional growth model with only the intercept and linear change in social anxiety symptoms was computed to examine the overall trajectory of social anxiety symptoms in the current sample. Model fit was good, *χ* ^2^ = 9.82, *p* = .08, CFI = .97, RMSEA = .08, SRMR = .07. When a quadratic term was entered, the model did not fit the data well, *χ* ^2^ = 4.45, *p* < .05, CFI = .98, RMSEA = .15, SRMR = .04. As such, we focus the analyses on the intercept and linear slope of social anxiety symptoms. There was a significant variance component for the intercept factor (*σ* ^2^ = 13.11, *SE* = 2.41, *z* = 5.43, *p* < .001), indicating significant variability in social anxiety symptoms at T1. The variance component for the slope factor was trending but did not reach statistical significance (*σ* ^2^ = 0.83, *SE* = 0.44, *z* = 1.89, *p* = .059), suggesting that there were no substantive individual differences in the shape of change across time. However, adding predictors can increase power to predict variability in linear trajectories.

### Aim 1: Peer and familial support/negative interactions as moderators of the association between BI and social anxiety

**FIGURE 1:**
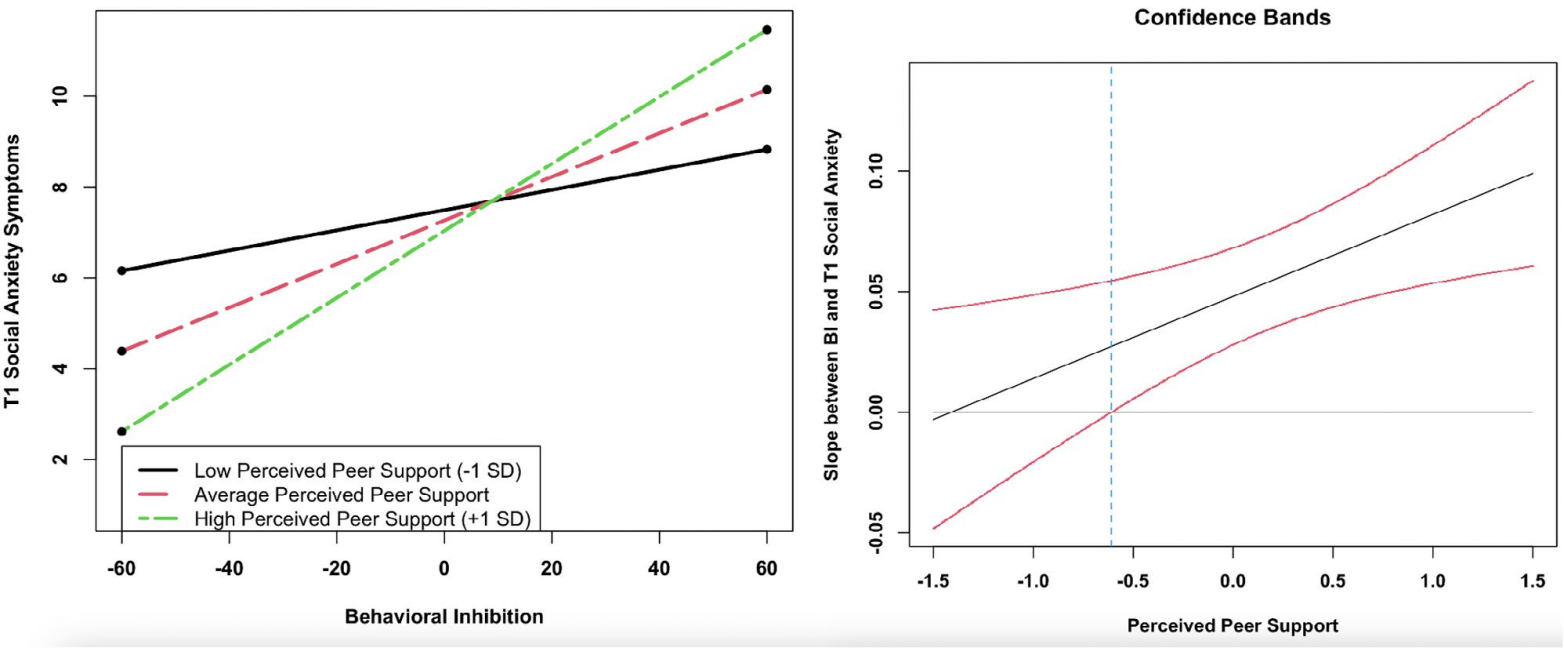
BI × perceived peer support predicting concurrent (T1) social anxiety symptoms in adolescent girls. *X*‐axis values represent mean‐centered scores. At low (−1 *SD*) perceived peer support, simple slope = .02, *SE* = .01, *p* = .14. At average perceived peer support, simple slope = .05, *SE* = .01, *p* < .001. At high (+1 *SD*) perceived peer support, simple slope = .07, *SE* = .01, *p* < .001. In the Johnson–Neyman plot, the effect of BI on social anxiety is significant for perceived peer support scores above −.61 units from the mean (observed score above 2.45), to the right of the vertical line. The Johnson–Neyman plot only includes the range of peer support values observed in the current sample.

Results of the moderation analyses predicting the intercept of social anxiety symptoms are presented in Table 2. There was a significant interaction between perceived peer support and BI on T1 social anxiety for adolescent girls (*β* = .29, *z* = 2.79, *p* = .005; Figure 1), and a likelihood ratio test indicated that this effect significantly differed for adolescent girls versus boys [Δ*χ* ^2^(1) = 5.72, *p* = .017]. Furthermore, likelihood ratio tests indicated that this coefficient significantly differed from those associated with interactions between BI and other peer and familial relationship qualities on T1 social anxiety symptoms for adolescent girls (*p*s < .05). The range of observed scores for perceived peer support for adolescent girls was 1.37 to 4.62, with a mean score of 3.06. The Johnson–Neyman region of significance indicated that the association between BI and social anxiety was significant for peer support ratings above −.61 units from the mean (observed score above 2.45), to the right of the vertical line presented in Figure 1. There were no other significant interactions between peer or familial relationship qualities and BI on concurrent social anxiety symptoms (*p*s > .05).

**TABLE 2:** Results of multiple‐group path models testing effects of peer and familial relationship qualities on the concurrent association between BI and social anxiety.

	Adolescent girls			Adolescent boys		
	Unstandardized *B* (*SE*)	*β*	*p*	Unstandardized *B* (*SE*)	*β*	*p*
**Step 1**						
BI	0.06 (.01)	.60	<.001	0.06 (.01)	.64	<.001
Peer support	−0.47 (.49)	−.11	.342	−0.46 (.74)	−.10	.530
Peer negative interactions	1.00 (.65)	.18	.126	−0.33 (.85)	−.07	.700
Familial support	−1.74 (.67)	−.34	.009	−.19 (1.05)	−.03	.858
Familial negative interactions	−.91 (.64)	−.16	.152	1.11 (1.15)	.18	.333
**Step 2**						
BI	0.05 (.01)	.51	<.001	0.05 (.02)	.54	.001
Peer support	−0.30 (.48)	−.07	.530	−0.79 (.74)	−.17	.284
Peer negative interactions	1.03 (.65)	.18	.115	−1.59 (1.07)	−.33	.138
Familial support	−2.07 (.65)	−.39	.001	0.05 (1.07)	.01	.965
Familial negative interactions	−1.02 (.63)	−.18	.104	1.81 (1.13)	.31	.109
BI × Peer support	**0.03 (.01)**	.**29**	.**005**	−0.02 (.02)	−.14	.355
BI × Peer negative interactions	−0.01 (.02)	−.05	.665	−0.07 (.04)	−.42	.084
BI × Familial support	−0.02 (.02)	−.09	.456	−0.01 (.03)	−.07	.712
BI × Familial negative interactions	−0.02 (.02)	−.10	.359	0.01 (.03)	.08	.674

*Note*: All predictors were mean‐centered both in interaction terms and when entered as main effects. Significant moderation effects are bolded.

**FIGURE 2:**
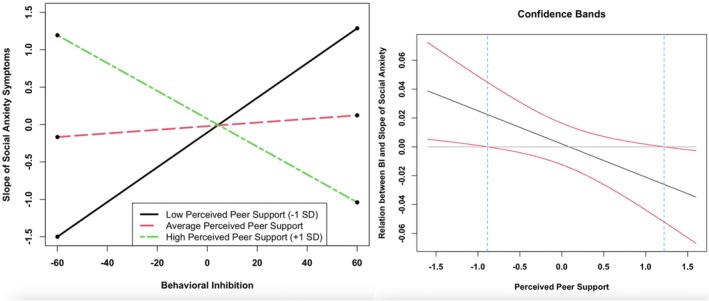
BI × perceived peer support predicting slope of social anxiety symptoms in adolescent girls. *X*‐axis values represent mean‐centered scores. At low (−1 *SD*) perceived peer support, simple slope = .02, *SE* = .01, *p* = .064. At average perceived peer support, simple slope = .00, *SE* = .01, *p* = .781. At high (+1 *SD*) perceived peer support, simple slope = −.02, *SE* = .01, *p* = .119. In the Johnson–Neyman plot, BI significantly predicts increasing social anxiety symptoms for perceived peer support scores below −0.89 units from the mean (observed score below 2.17). BI significantly predicts decreasing social anxiety symptoms for perceived peer support scores above 1.22 units from the mean (observed score above 4.28). The Johnson–Neyman plot only includes the range of peer support values observed in the current sample.

Turning to moderators of the association between BI and social anxiety symptom trajectories (Table 3), we observed a significant interaction between perceived peer support and the slope of social anxiety symptoms across time for adolescent girls (*β* = −.59, *z* = −2.48, *p* = .013; Figure 2). Model fit improved when this parameter was allowed to differ for adolescent girls versus boys, although the likelihood ratio test did not reach statistical significance [Δ*χ* ^2^(1) = 3.68, *p* = .055]. Furthermore, likelihood ratio tests indicated that this coefficient significantly differed from those associated with interactions between BI and other peer and familial relationship qualities on social anxiety symptom trajectories in adolescent girls (*p*s < .05). The Johnson–Neyman region of significance showed that BI significantly predicted increasing social anxiety symptoms when perceived peer support was low (observed score below 2.17). Alternatively, BI significantly predicted decreasing social anxiety symptoms when perceived peer support was high (observed score above 4.28).

**TABLE 3:** Results of multiple‐group path models testing effects of peer and familial relationship qualities on the association between BI and the slope of social anxiety symptoms across adolescence.

	Adolescent girls			Adolescent boys		
	Unstandardized *B* (*SE*)	*β*	*p*	Unstandardized *B* (*SE*)	*β*	*p*
**Step 1**						
BI	0.00 (.01)	.09	.704	0.00 (.01)	.00	.993
Peer support	0.11 (.37)	.09	.766	0.19 (.36)	.14	.609
Peer negative interactions	−0.09 (.49)	−.06	.858	0.72 (.48)	.54	.132
Familial support	0.21 (.48)	.14	.667	0.36 (.47)	.22	.449
Familial negative interactions	0.55 (.47)	.35	.240	0.59 (.60)	.37	.321
**Step 2**						
BI	0.00 (.01)	.07	.756	0.00 (.01)	.05	.852
Peer support	−0.09 (.40)	−.06	.827	0.50 (.37)	.29	.178
Peer negative interactions	−0.07 (.48)	−.04	.886	0.02 (.65)	.01	.980
Familial support	0.45 (.50)	.26	.364	0.07 (.47)	.03	.888
Familial negative interactions	0.74 (.48)	.39	.121	1.16 (.66)	.55	.079
BI × Peer support	**−0.02 (.01)**	**−.59**	.**013**	0.00 (.01)	.06	.809
BI × Peer negative interactions	−0.01 (.01)	−.20	.358	−0.02 (.02)	−.30	.429
BI × Familial support	0.02 (.01)	.26	.285	0.02 (.02)	.29	.247
BI × Familial negative interactions	0.02 (.01)	.33	.145	**0.05 (.02)**	.**84**	.**010**

*Note*: All predictors were mean‐centered both in interaction terms and when entered as main effects. Significant moderation effects are bolded.

**FIGURE 3:**
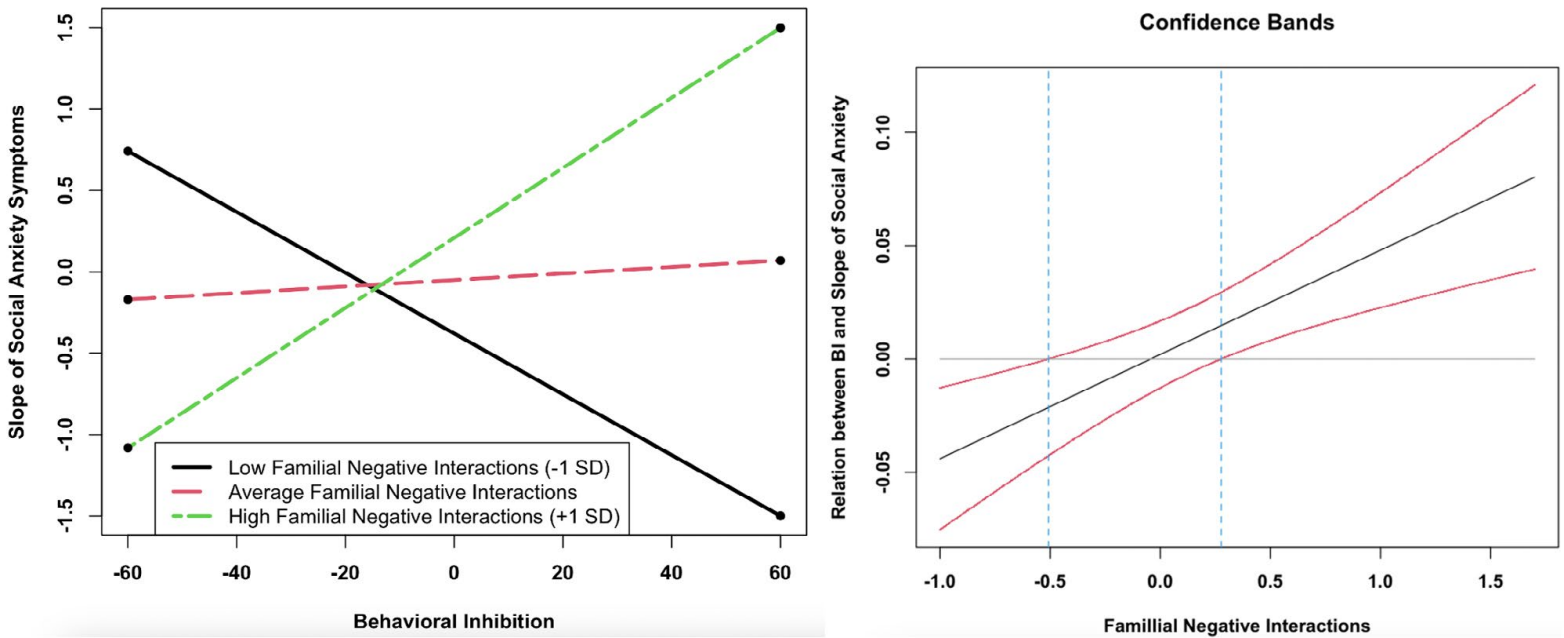
BI × perceived familial negative interactions predicting slope of social anxiety symptoms in adolescent boys. *X*‐axis values represent mean‐centered scores. At low (−1 *SD*) perceived familial negative interactions, simple slope = −.02, *SE* = .01, *p* = .036. At average perceived familial negative interactions, simple slope = .00, *SE* = .01, *p* = .78. At high (+1 *SD*) perceived familial negative interactions, simple slope = .03, *SE* = .01, *p* = .003. In the Johnson–Neyman plot, BI significantly predicts increasing social anxiety symptoms for familial negative interactions scores above 0.28 units from the mean (observed score above 2.26). BI significantly predicts decreasing social anxiety symptoms for familial negative interactions scores below −0.51 units from the mean (observed score below 1.47). The Johnson–Neyman plot only includes the range of familial negative interactions observed in the current sample.

We also observed a significant interaction between perceived familial negative interactions and BI on the slope of social anxiety symptoms for adolescent boys (*β* = .84, *z* = 2.56, *p* = .010; Figure 3). A likelihood ratio test indicated that this effect did not significantly differ for adolescent boys versus girls [Δ*χ* ^2^(1) = 1.75, *p* = .186]. However, this coefficient did significantly differ from those associated with interactions between BI and other peer and familial relationship qualities on social anxiety symptom trajectories in adolescent boys (*p*s < .05). The range of observed scores for familial negative interactions for adolescent boys was 1.00 to 3.79, with a mean score of 1.98. The Johnson–Neyman region of significance showed that BI significantly predicted increasing social anxiety symptoms when familial negative interactions scores were high (observed score above 2.26). BI significantly predicted decreasing social anxiety symptoms when familial negative interactions scores were low (observed score below 1.47). There were no other significant interactions between peer or familial relationship qualities and BI on trajectories of social anxiety symptoms (*p*s > .05).

### Aim 2: Indirect effects of BI on social anxiety through peer and familial relationship qualities

In line with our second aim, we next examined the indirect effects of BI on social anxiety through peer and familial relationship qualities. We examined the indirect effect of BI on social anxiety symptoms measured at the subsequent timepoint (T2), controlling for prior levels of social anxiety symptoms, in order to establish temporal separation of BI and social anxiety. As seen in Table 4, we found a significant indirect effect of BI on social anxiety through perceived familial support for adolescent girls (*β* = .10, *z* = 2.15, *p* = .031), but not for adolescent boys (*β* = .02, *z* = 0.44, *p* = .66). Likelihood ratio tests suggested that both the *a* and *b* paths differed for adolescent girls versus boys, although these did not reach statistical significance [Δ*χ* ^2^(1) = 2.72, *p* = .099 for *a* path; Δ*χ* ^2^(1) = 3.13, *p* = .077 for *b* path]. BI was associated with lower perceived familial support at T1 (*β* = −.32, *z* = −3.70, *p* < .001), which in turn predicted higher social anxiety at T2 (*β* = −.32, *z* = −2.66, *p* = .008). No other indirect effects were significant. Full results for all models of indirect effects are presented in Table 4.

**TABLE 4:** Indirect effects of peer and familial relationship qualities on the longitudinal association between BI and social anxiety.

Variable/Effect	Adolescent girls				Adolescent boys			
	*B* (*SE*)	*β*	*z*	*p*	*B* (*SE*)	*β*	*z*	*p*
*Mediator: Familial support*								
Familial support to SA	**−1.39 (.52)**	**−.32**	**−2.66**	.**008**	−.45 (.78)	−.10	−0.58	.56
BI to familial support	**−.34 (.09)**	**−.32**	**−3.70**	**<.001**	−.15 (.11)	−.16	−1.36	.18
Indirect	.**47 (.22)**	.**10**	**2.15**	.**031**	.07 (.15)	.02	0.44	.66
*Mediator: Peer support*								
Peer support to SA	−.27 (.54)	−.06	−0.51	.61	−.14 (.13)	−.10	−0.69	.49
BI to peer support	−.07 (.12)	−.07	−0.55	.58	−.01 (.16)	−.01	−.08	.94
Indirect	.02 (.08)	.00	0.22	.82	−.01 (.21)	.00	−0.06	.95
*Mediator: Peer negative int*.								
Peer negative int. to SA	.64 (.63)	.13	1.01	.31	.51 (1.15)	.12	0.44	.66
BI to peer negative int.	.17 (.09)	.18	1.90	.06	.01 (.09)	.01	0.08	.94
Indirect	.11 (.13)	.02	0.82	.41	.00 (.11)	.00	0.03	.97
*Mediator: Familial negative int*.								
Familial negative int. to SA	−.26 (.50)	−.06	−0.52	.60	.95 (.86)	.23	1.10	.27
BI to familial negative int.	.11 (.11)	.11	1.05	.29	.20 (.13)	.20	1.48	.14
Indirect	−.03 (.08)	−.01	−0.36	.72	.19 (.23)	.05	0.81	.42

*Note*: Bold text indicates significant effects; SA = social anxiety at T2. T1 social anxiety symptoms were covaried.

### Post hoc analyses

To examine whether the observed effects were specific to social anxiety, we repeated the analyses using generalized anxiety symptoms as the dependent variable. The significance and direction of all observed moderation effects, with the exception of the interaction between BI and perceived peer support predicting the slope of social anxiety symptoms for adolescent girls, replicated when generalized anxiety was entered as the outcome (see Tables S1–S3 for the full statistics). Although the indirect effect of BI on generalized anxiety symptoms through perceived familial support was not statistically significant, the beta coefficients were comparable (*β* = .10 for social anxiety and *β* = .12 for generalized anxiety among female adolescents), supporting the replicability of this finding. Of note, social and generalized anxiety were highly comorbid in the current sample, with 86.7% of participants who met the clinical cutoff for SAD symptoms also reporting clinically significant GAD symptoms at the same timepoint.

We also examined potential curvilinear effects of familial support on the BI‐social anxiety association, given prior work showing this pattern in young children (Kiel et al., 2016). Specifically, we tested the interaction between BI and a quadratic term of familial support in the prediction of concurrent social anxiety (*β* = −.23, *z* = −1.43, *p* = .153 for girls; *β* = −.20, *z* = −1.04, *p* = .297 for boys) and social anxiety trajectories (*β* = −.35, *z* = −0.95, *p* = .344 for girls; *β* = .15, *z* = 0.65, *p* = .515 for boys), which were all nonsignificant. For indirect effects, we entered a quadratic effect of familial support on subsequent social anxiety (path *b*), which was again nonsignificant for boys (*β* = 0.03, *z* = 0.86, *p* = .391) and girls (*β* = 0.02, *z* = 0.51, *p* = .610).

## DISCUSSION

In the current study, we used a longitudinal, multi‐informant design to examine (1) the moderating role of perceived familial and close peer relationship qualities on associations between BI, concurrent social anxiety, and social anxiety symptom trajectories across adolescence and (2) indirect effects of BI on later social anxiety through close peer and familial relationship qualities. For adolescent girls, higher perceived support from close peers strengthened the association between BI and concurrent social anxiety symptoms. Additionally, perceived support from close peers interacted with BI to predict trajectories of social anxiety symptoms among adolescent girls, such that BI predicted increasing social anxiety for those with lower perceived support from peers and decreasing social anxiety for those with higher perceived peer support. BI indirectly predicted later social anxiety through low perceived familial support for adolescent girls but unexpectedly not through close peer relationship qualities. For adolescent boys higher in BI, high levels of perceived negative interactions with family members predicted increasing social anxiety symptoms across time, whereas low levels of perceived familial negative interactions predicted decreasing social anxiety symptoms. To our knowledge, this study was among the first to examine proximal family and peer influences on the association between adolescent BI and social anxiety symptomatology. Results provide support for risk and protective factors that shape social anxiety trajectories across this vulnerable developmental period for inhibited adolescents.

The relation between close peer support and social anxiety, particularly for BI youth, is complex and understudied, representing a critical contribution of the current study to existing models of social anxiety risk. Our finding that more inhibited adolescent girls who reported greater support from close peers concurrently reported higher social anxiety symptoms is consistent with other work showing that, in some cases, friendships can serve as a risk factor for inhibited youth (Barstead et al., 2018; Rubin et al., 2006). There are a couple of potential mechanisms that may explain this association. First, inhibited adolescent girls may experience a heightened desire for peer approval (Coplan et al., 2004; Guyer et al., 2014), which could increase perceived support from close peers while simultaneously facilitating social anxiety. Consistent with this prediction, empirical work has shown that sensitivity to peer approval and the tendency to change one's thoughts and behaviors to align with peers is associated with greater social anxiety symptoms (Weymouth et al., 2019; Weymouth & Buehler, 2018). Second, it could be that adolescent girls high in BI or anxiety symptoms elicit forms of support from their close peers, such as accommodating behaviors like talking for them or going with them to new clubs or activities, that facilitate avoidance and exacerbate concurrent anxiety symptoms. Such accommodation may impede youth who are higher in BI from developing the necessary socio‐cognitive skills to navigate social situations independently, consequently increasing or maintaining their social anxiety symptoms.

Interestingly, the opposite pattern emerged when we examined trajectories of social anxiety symptoms across adolescence. Specifically, more inhibited girls reported increasing social anxiety symptoms when perceived support from peers was low and reported decreasing social anxiety symptoms when perceived peer support was high. This effect was specific to trajectories of social anxiety, versus generalized anxiety symptoms. Of note, most adolescents who were higher in BI entered the study with high levels of social anxiety symptoms that were relatively stable across the study period, suggesting that these findings represent a subset of youth who were more sensitive to socio‐contextual influences specific to adolescence. This could suggest that the developmental timing of social anxiety onset shapes how risk is conferred. Specifically, the cross‐sectional findings could reflect social processes that emerge prior to adolescence and serve to maintain early‐emerging social anxiety symptoms across development. On the contrary, the longitudinal findings could reflect socio‐contextual risk and protective factors that influence social anxiety development specifically during adolescence. Consistent with this interpretation, peer acceptance and integration take on increasing importance during adolescence, and many empirical studies show that peer support directly relates to positive adolescent mental health outcomes (Roach, 2018). Thus, while high peer support, potentially in the form of over‐accommodation, may contribute to social anxiety development in middle to late childhood, lower perceived support from peers may be more relevant to social anxiety risk during adolescence. This is in line with our recent conceptual model of internalizing development, which highlights how social threat sensitivity may manifest differently across childhood and adolescence in ways that uniquely shape peer relationships and internalizing risk among temperamentally fearful youth (Politte‐Corn et al., 2026). Future work could test this prediction more directly by examining whether there are qualitative differences in peer support that shape social anxiety development in childhood versus adolescence.

We also found that perceived familial support mediated, but did not moderate, the association between BI and social anxiety for adolescent girls. Specifically, BI was associated with lower perceived familial support, which in turn predicted higher social anxiety symptoms 1 year later. Research on familial support and social anxiety risk in adolescence is limited and mixed, such that some forms of support from family members may be protective (Boulton & Macaulay, 2022) while overprotective tendencies may increase risk (Nelemans et al., 2020). Importantly, our composite measure of familial support captured several relationship qualities, including companionship, intimate disclosure, and nurturance/affection. Our finding that BI was associated with lower perceived familial support may be consistent with studies documenting bidirectional relations between BI and oversolicitous/affectionate caregiving (Buss et al., 2021; Kiel et al., 2021), if adolescents do not perceive this behavior as supportive. However, given that our BI and familial support measures were separated only by a few months on average, we cannot make strong claims about the directionality of this association. Furthermore, we posited that while overprotective and accommodating parenting may increase social anxiety risk, other types of support are likely protective for BI adolescents, which is consistent with the indirect effect we observed. Specificity in this indirect effect for female adolescents suggests that these protective forms of support from family members, likely characterized by engagement with youths' emotions and appropriate guidance (Miller‐Slough & Dunsmore, 2016), may be particularly impactful in reducing later social anxiety for female adolescents higher in BI relative to more inhibited male adolescents.

For more inhibited adolescent boys, greater perceived negative interactions within the family predicted increasing social anxiety symptoms and lower perceived negative interactions within the family predicted decreasing social anxiety symptoms. Given higher prevalence rates of anxiety in females compared with males, much of the anxiety literature has focused on adolescent girls being at higher risk (e.g., Greco & Morris, 2005; Pickering et al., 2020; Rose & Rudolph, 2006). However, numerous studies have demonstrated that, developmentally, BI boys are at higher risk for anxiety‐related outcomes than BI girls (Gest, 1997; Henderson et al., 2001; Kagan et al., 1998; Zhou et al., 2022), potentially due to socio‐cultural norms related to shyness and fear. Within the familial context, prior studies have shown that parents respond to boys' fearful behaviors with greater concern, intrusiveness, and protective parenting compared with girls (Kiel et al., 2016; Park et al., 1997), and this oversolicitousness predicts greater risk for the development of social anxiety among fearful youth (Kiel & Buss, 2014). In the current study, intrusive or overprotective parenting may have manifested as greater perceived negative interactions within the family among adolescent boys, contributing to prospective increases in social anxiety, or prospective decreases in social anxiety when intrusiveness and conflict was low. Alternatively, prior work with adolescents found that boys negatively appraised interparental conflict regardless of other protective factors in the family, whereas adolescent girls were less prone to negatively appraise interparental conflict when other familial protective factors (e.g., cohesion) were present (Fosco et al., 2021). As such, it could be that adolescent boys benefit less from supportive interactions within the family and consequently are more vulnerable to familial interactions perceived as negative, which is consistent with our finding that perceived familial support was protective specifically for adolescent females.

Finally, our sensitivity analyses indicated that most findings replicated when generalized anxiety was examined as the outcome variable, suggesting that these effects characterized risk for anxiety more broadly. Of note, SAD and GAD symptoms were highly comorbid in our sample, suggesting that these findings are likely capturing risk within the same subset of youth rather than different risk subtypes. There is a large and robust literature linking BI temperament and the development of social anxiety in particular (e.g., Chronis‐Tuscano et al., 2009; Clauss & Blackford, 2012; Hirshfeld‐Becker et al., 2007). However, some studies do find associations between BI and generalized anxiety (Bourdon et al., 2019; Fox et al., 2022; Kagan & Snidman, 1999), particularly when anxiety symptoms are assessed during adolescence or later. As such, BI may show greater specificity in predicting social withdrawal and anxiety earlier in development, then predict a broader range of anxiety‐related problems by adolescence.

### Limitations and future directions

While the present study has several strengths, there are also limitations that should guide future work. First, there were limitations to our measurement and analysis of peer and familial relationships qualities. Because we used self‐report measures, our findings reflect the role of *perceived*, rather than objective, relationship qualities in shaping associations between BI and social anxiety. However, while subject to reference bias, self‐report measures are in many cases highly predictive of objectively measured outcomes (Duckworth & Yeager, 2015; Lucas & Baird, 2006), and understanding the role of perceived relationship qualities in social anxiety risk is informative in and of itself. Relatedly, we showed that peer and familial effects on anxiety significantly differed from each other, but these differences could have been due to varying measurement properties across the peer and familial measures. Additionally, we predicted that familial support at both low and high levels (i.e., indicating oversolicitous/overprotective parenting) could confer risk for social anxiety among BI youth, but our measure of familial support did not directly assess overcontrol or problematic accommodation behaviors. To more directly compare to prior work showing a curvilinear association between parental support/encouragement and anxiety in young children (Kiel et al., 2016), future studies could directly measure over‐accommodation versus low levels of support among parents of BI adolescents. Turning to our analytic strategy, we modeled familial and peer influences separately, but in reality, adolescents are developing embedded in a network of relationships across peer and familial contexts. As such, future work should examine potential additive or multiplicative effects of support or negative interactions across relationships or use person‐centered methods that can capture how peer and familial relationship qualities hold together within individuals. It will also be important for future work to examine bidirectional effects of social anxiety and peer and familial relationship qualities across adolescence, as anxious adolescents may elicit different types of social interactions.

Second, we assessed BI during adolescence, so it is possible that our BI measure was confounded by the onset of social anxiety symptoms. However, despite phenotypic similarities in some associated behaviors (e.g., social avoidance, fear of negative evaluation), many studies suggest that BI and social anxiety are distinct constructs with unique biobehavioral profiles (e.g., Klein & Mumper, 2018; Pérez‐Edgar & Guyer, 2014). Indeed, our prior work using a subset of the current sample showed that BI and social anxiety are characterized by unique neural correlates (Politte‐Corn et al., 2025). Third, our sample was predominantly White. Given our emphasis on socio‐contextual risk and resilience factors, it is likely that these processes differ across cultures (Heinrichs et al., 2006), and it is critical to examine the impact of familial and peer relationship qualities on social anxiety risk in more diverse samples. However, participating families did report a wide range of familial income and education levels, which is a strength of the current study and adds to existing research. Finally, our follow‐up sample was significantly smaller than planned due to the COVID‐19 pandemic, which may have impacted our ability to detect small effect sizes in the longitudinal models. It is possible that there were lingering effects of the pandemic context on our main constructs, such as overall increases in social anxiety or changes in peer or familial dynamics, which could have impacted the pattern of findings.

## CONCLUSION

The current study is an important step in characterizing the socio‐contextual experiences in early adolescence that shape trajectories of social anxiety for youth at temperamental risk. Findings indicated that perceived support from close peers, but not peer negative interactions or familial support, increased social anxiety risk for adolescent girls higher in BI. Furthermore, BI interacted with perceived familial negative interactions to predict social anxiety symptoms trajectories, specifically for adolescent boys. Finally, lower perceived familial support, but not close peer relationship qualities, mediated the effect of BI on later social anxiety for adolescent girls. These findings provide new insight into risk factors for social anxiety among BI youth and highlight several potential developmental pathways to social anxiety to guide future work.

## AUTHOR CONTRIBUTIONS

**Sarah Myruski:** Methodology; writing – review and editing; supervision; conceptualization. **Kristin A. Buss:** Conceptualization; methodology; writing – review and editing; supervision; funding acquisition. **Madison Politte‐Corn:** Conceptualization; methodology; formal analysis; writing – original draft.

## FUNDING INFORMATION

Data collection was supported by the National Institute of Mental Health, Grant R01‐MH114974, awarded to KAB. MPC was supported by the National Center for Advancing Translational Sciences (Grant TL1 TR002016), in the data analysis and writing of this manuscript. KB was supported by the McCourtney Professorship in Children, Work, and Families and by the Social Science Research Institute of Penn State University in the writing of this manuscript.

## CONFLICT OF INTEREST STATEMENT

The authors have no conflicts of interest to disclose.

## ETHICS STATEMENT

The Institutional Review Board at Pennsylvania State University approved the most recent study procedures on May 23, 2022 (STUDY00009742). All parents of adolescents provided informed consent, and all adolescents provided informed assent prior to participation in this study.

## Supporting information


**Table S1.** Results of multiple‐group path models testing effects of peer and familial relationship qualities on the concurrent association between BI and generalized anxiety.
**Table S2**. Results of multiple‐group path models testing effects of peer and familial relationship qualities on the association between BI and the slope of generalized anxiety symptoms across adolescence.
**Table S3**. Indirect effects of peer and familial relationship qualities on the association between BI and T2 generalized anxiety.

## Data Availability

The data that support the findings of this study are openly available in the National Institutes of Health National Data Archive (NIH‐NDA).
