# Supplementary material for: Peer and familial influences on the association between behavioral inhibition and trajectories of social anxiety symptoms across adolescence
Source: J Res Adolesc. 2026 May 15;36:e70194. doi: 10.1111/jora.70194 (PMC13179430; doi:10.1111/jora.70194)
Supplement: Supplementary file 1 — Table S1. Results of multiple‐group path models testing effects of peer and familial relationship qualities on the concurrent association between BI and generalized anxiety. Table S2. Results of multiple‐group path models testing effects of peer and familial relationship qualities on the association between BI and the slope of generalized anxiety symptoms across adolescence. Table S3. Indirect effects of peer and familial relationship qualities on the association between BI and T2 generalized anxiety. [file JORA-36-0-s001.docx]

**Table S1**

*Results of multiple-group path models testing effects of peer and familial relationship qualities on the concurrent association between BI and generalized anxiety.*

|  |  | **Adolescent Girls** | | | **Adolescent Boys** | | |
| --- | --- | --- | --- | --- | --- | --- | --- |
|  |  | **Unstandardized B (SE)** | ***β*** | ***p*** | **Unstandardized B (SE)** | ***β*** | ***p*** |
| **Step 1** | BI | 0.02 (.01) | .23 | .098 | 0.05 (.02) | .56 | <.001 |
|  | Peer support | 0.89 (.66) | .18 | .179 | 0.78 (.72) | .17 | .280 |
|  | Peer negative interactions | 1.50 (.88) | .24 | .086 | 0.10 (.87) | .02 | .910 |
|  | Familial support | -1.35 (.87) | -.24 | .122 | -1.25 (1.03) | -.22 | .227 |
|  | Familial negative interactions | 1.00 (.86) | .16 | .240 | 0.40 (1.15) | .07 | .729 |
| **Step 2** |  |  |  |  |  |  |  |
|  | BI | 0.00 (.02) | .04 | .800 | 0.04 (.02) | .45 | .016 |
|  | Peer support | 1.25 (.64) | .25 | .052 | 0.45 (.70) | .11 | .519 |
|  | Peer negative interactions | 2.07 (.89) | .32 | .020 | -1.33 (1.05) | -.33 | .203 |
|  | Familial support | -1.89 (.87) | -.32 | .029 | -0.48 (1.03) | -.10 | .640 |
|  | Familial negative interactions | 0.82 (.85) | .13 | .331 | 1.11 (1.10) | .22 | .315 |
|  | BI X Peer support | **0.03 (.02)** | **.26** | **.040** | -0.02 (.02) | -.16 | .349 |
|  | BI X Peer negative interactions | -0.05 (.03) | -.25 | .072 | -0.08 (.04) | -.55 | .063 |
|  | BI X Familial support | 0.00 (.03) | .01 | .947 | 0.02 (.03) | .12 | .616 |
|  | BI X Familial negative interactions | 0.01 (.02) | .08 | .552 | -0.00 (.03) | -.04 | .884 |

*Note:* All predictors were mean-centered both in interaction terms and when entered as main effects. Significant moderation effects are bolded.

**Table S2**

*Results of multiple-group path models testing effects of peer and familial relationship qualities on the association between BI and the slope of generalized anxiety symptoms across adolescence.*

|  |  | **Adolescent Girls** | | | **Adolescent Boys** | | |
| --- | --- | --- | --- | --- | --- | --- | --- |
|  |  | **Unstandardized B (SE)** | ***β*** | ***p*** | **Unstandardized B (SE)** | ***β*** | ***p*** |
| **Step 1** | BI | -0.00 (.01) | -.04 | .904 | 0.00 (.01) | .05 | .861 |
|  | Peer support | -0.64 (.39) | -.71 | .098 | -0.29 (.42) | -.20 | .488 |
|  | Peer negative interactions | -1.33 (.51) | -1.14 | .009 | 0.61 (.56) | .40 | .281 |
|  | Familial support | 0.23 (.51) | .21 | .656 | 0.93 (.53) | .50 | .080 |
|  | Familial negative interactions | 0.39 (.51) | .34 | .446 | 0.87 (.69) | .48 | .206 |
| **Step 2** |  |  |  |  |  |  |  |
|  | BI | 0.00 (.01) | .05 | .887 | 0.02 (.01) | .51 | .130 |
|  | Peer support | -0.79 (.45) | -.70 | .077 | -0.30 (.43) | -.21 | .488 |
|  | Peer negative interactions | -1.52 (.52) | -1.04 | .004 | 0.59 (.72) | .41 | .409 |
|  | Familial support | 0.33 (.55) | .25 | .548 | 0.82 (.56) | .46 | .144 |
|  | Familial negative interactions | 0.27 (.54) | .19 | .620 | 0.73 (.78) | .41 | .347 |
|  | BI X Peer support | 0.00 (.01) | .08 | .827 | 0.01 (.01) | .26 | .424 |
|  | BI X Peer negative interactions | 0.02 (.02) | .40 | .212 | 0.00 (.03) | .03 | .951 |
|  | BI X Familial support | 0.01 (.02) | .18 | .600 | **0.04 (.02)** | **.82** | **.038** |
|  | BI X Familial negative interactions | 0.01 (.01) | .20 | .558 | **0.05 (.02)** | **1.18** | **.018** |

*Note:* All predictors were mean-centered both in interaction terms and when entered as main effects. Significant moderation effects are bolded.

| **Table S3**  Indirect effects of peer and familial relationship qualities on the association between BI and T2 generalized anxiety. | | | | | | | | | | |  |  |
| --- | --- | --- | --- | --- | --- | --- | --- | --- | --- | --- | --- | --- |
|  |  | **Adolescent Girls** | | | | | | **Adolescent Boys** | | | |  |
| *Mediator: Familial support* | *Variable / Effect* | B (SE) | *β* | | *z* | | *p* | B (SE) | *β* | *z* | *p* |  |
|  | Familial support to GA | **-.31 (.12)** | **-.37** | | **-2.55** | | **.01** | -.19 (.12) | -.20 | -1.62 | .11 |  |
|  | BI to familial support | **-.34 (.09)** | **-.32** | | **-3.70** | | **<.001** | -.15 (.11) | -.16 | -1.36 | .18 |  |
|  | Indirect | .11 (.06) | .12 | | 1.67 | | .10 | .03 (.03) | .03 | 0.88 | .38 |  |
| *Mediator: Peer support* | *Variable / Effect* | B (SE) | *β* | | *z* | | *p* | B (SE) | *β* | *z* | *p* |  |
|  | Peer support to GA | -.06 (.12) | -.07 | | -0.49 | | .62 | .19 (.20) | .21 | 0.95 | .34 |  |
|  | BI to peer support | -.07 (.12) | -.07 | | -0.55 | | .58 | -.01 (.16) | -.01 | -.08 | .94 |  |
|  | Indirect | .00 (.02) | .00 | | 0.22 | | .83 | .00 (.05) | .00 | -0.05 | .96 |  |
| *Mediator: Peer negative int.* | *Variable / Effect* | B (SE) | *β* | | *z* | | *p* | B (SE) | *β* | *z* | *p* |  |
|  | Peer negative int. to GA | .14 (.17) | .14 | | 0.82 | | .41 | .36 (.21) | .38 | 1.71 | .09 |  |
|  | BI to peer negative int. | .17 (.09) | .18 | | 1.90 | | .06 | .01 (.09) | .01 | 0.08 | .94 |  |
|  | Indirect | .02 (.04) | .03 | | 0.65 | | .52 | .00 (.04) | .00 | 0.08 | .94 |  |
| *Mediator: Familial negative int.* | *Variable / Effect* | B (SE) | *β* | *z* | | *p* | | B (SE) | *β* | *z* | *p* |  |
|  | Familial negative int. to GA | .17 (.14) | .19 | 1.21 | | .23 | | **.34 (.16)** | **.37** | **2.09** | **.04** |  |
|  | BI to familial negative int. | .11 (.11) | .11 | 1.05 | | .29 | | .20 (.13) | .20 | 1.48 | .14 |  |
|  | Indirect | .02 (.03) | .02 | 0.64 | | .52 | | .07 (.06) | .07 | 1.08 | .28 |  |
| *Note.* Bold text indicates significant effects; GA = generalized anxiety at T2. | | | | | | | | | | | | |
